# Assessing Array-Type Differences in Cochlear Implant Users Using the Panoramic ECAP Method

**DOI:** 10.1097/AUD.0000000000001673

**Published:** 2025-05-22

**Authors:** Charlotte Garcia, Robert P. Carlyon

**Affiliations:** Cambridge Hearing Group, Medical Research Council Cognition & Brain Sciences Unit, University of Cambridge, Cambridge, United Kingdom

**Keywords:** Cochlear Implant, Panoramic ECAP, Current Spread, Array Type, Electrode-Modiolus Distances

## Abstract

**Objectives:**

Cochlear-implant companies manufacture devices with different electrode array types. Some arrays have a straight geometry designed for minimal neuronal trauma, while others are precurved and designed to position the electrodes closer to the cochlear neurons. Due to their differing geometries, it’s possible that the arrays are not only positioned differently inside the cochlea, but also produce different patterns of the spread of current and of neural excitation. The Panoramic ECAP method (PECAP) provides detailed estimates of peripheral neural responsiveness and current spread for individual patients along the length of the cochlea. These estimates were assessed as a function of electrode position and array type, providing a normative dataset useful for identifying unusual patterns in individual patients.

**Design:**

ECAPs were collected from cochlear-implant users using the forward-masking artefact-reduction technique for every combination of masker and probe electrode at the most comfortable level. Data were available for 91 ears using Cochlear © devices, and 53 ears using Advanced Bionics © devices. The Cochlear users had straight arrays (Slim Straight, CI-22 series, n=35), or one of two precurved arrays (Contour Advance, CI-12 series, n=43, or Slim Modiolar, CI-32 series, n=13). Computed Tomography (CT) scans were also available for 41 of them, and electrode-modiolus distances were calculated. The Advanced Bionics users had one of two straight arrays (1J, n=9 or SlimJ, n=20), or precurved arrays (Helix, n=4 or Mid-Scala, n=20). The ECAPs were submitted to the PECAP algorithm to estimate current spread and neural responsiveness along the length of the electrode array for each user. A Linear Mixed Effects Model was used to determine whether there were statistically significant differences between different array types and/or for different electrodes, both for the PECAP estimates of current spread and neural responsiveness, as well as for the available electrode-modiolus distances. Correlations were also conducted between PECAP’s estimate of current spread and the electrode-modiolus distances.

**Results:**

For Cochlear users, significant effects of array type (*p* = 0.001) and of electrode (*p* < 0.001) were found on the PECAP’s current-spread estimate, as well as a significant interaction (*p* = 0.006). Slim Straight arrays had wider overall current spread than both the precurved arrays (Contour Advance and Slim Modiolar). The interaction revealed the strongest effect at the apex. A significant correlation between PECAP’s current-spread estimate and the electrode-modiolus distances was also found across subjects (r = 0.516, *p* < 0.001). No effect of array type was found on PECAP’s estimate of current spread for the Advanced Bionics users (*p* = 0.979).

**Conclusions:**

These results suggest that for users of the Cochlear device, precurved electrode arrays show narrower current spread within the cochlea than those with lateral-wall electrode arrays, with the strongest effect present at the apex. No corresponding effects of array type were found in the Advanced Bionics device. This could have implications for device selection in clinical settings, although the authors underscore that this is a post-hoc analysis and does not demonstrate a causal link wherein device selection can be expected to give rise to specific neural-excitation patterns.

## Introduction

Cochlear implants (CIs) are neuro-prosthetic devices that enable auditory perception for severe-to-profoundly deaf people by directly stimulating the auditory nerve. Many CI users obtain good speech perception with their devices, but there is a lot of variability in outcomes and many struggle to understand speech, especially in challenging listening conditions (Firszt et al., 2004; Friesen et al., 2001). Not only do CI users make use of a limited number of spectral channels (Archer-Boyd et al., 2018; Goehring et al., 2014; White et al., 1984) compared to those with acoustic hearing (Moore & Carlyon, 2006; Moore & Peters, 1992), but the number of independent spectral channels varies widely and may be further limited by poor electrode-neuron interfaces (Bierer, 2010; Chatterjee & Shannon, 1998; Nelson et al., 1995).

When designing cochlear implant electrode arrays, an ideal scenario is one that places the electrodes as closely as possible to the surviving neural tissue without causing any additional trauma. The further away electrodes are from neurons the higher the current level required to stimulate them (Davis et al., 2016; Lee et al., 2019), and the more likely it is that adjacent channels will stimulate overlapping areas (Wagner et al., 2023), thereby reducing the number of independent perceptual channels (Ramos de Miguel et al., 2018; Zhou et al., 2023). However, the closer the electrodes are to the neural tissue, the more likely it is that they will cause trauma to the surviving neurons during insertion, potentially also reducing the number of independent perceptual channels due to localized “dead” regions (Dhanasingh & Jolly, 2017). While there are now some automated methods being developed for electrode insertion (Gawęcki et al., 2022; Jia et al., 2021; Rau et al., 2020), cochlear implantation is largely still a manual process done by an Ear, Nose, and Throat (ENT) surgeon, and involves removal of bone and subsequent insertion of a tiny electrode array into the cochlea under a microscope, a delicate process that requires precision. This must also be considered when designing electrode arrays to optimize both electrode placement and neuronal survival, with the ultimate aim of providing the most detailed perceivable signal and hence the best chance of obtaining good speech perception.

There are two broad categories of electrode array designs. One uses precurved arrays that are often designed to produce a perimodiolar placement, such that the electrodes are close to the neurons and minimize channel interaction. In contrast, straight or lateral-wall electrode arrays are designed to avoid neural trauma during cochlear implant surgery (Dhanasingh & Jolly, 2017; Ertas et al., 2022). The straight arrays provide an advantage over the precurved ones as the latter show higher incidences of cross-scala translocation (O’Connell et al., 2016; Wanna et al., 2014), presumed to cause neuronal trauma, although this doesn’t necessarily translate to poorer speech performance (Liebscher et al., 2021; Perkins et al., 2022). The precurved arrays show advantages over the straight ones in terms of lower impedances (Holder et al., 2019; Velandia et al., 2020), lower ECAP thresholds (Jeong et al., 2015; Telmesani & Said, 2015), lower perceptual threshold (T) and comfort (C) levels (Jeong et al., 2015; Lee et al., 2019) (although this benefit may be specific to the basal region (Christov et al., 2019)), narrower electrode-level electrical spread (Wagner et al., 2023), and superior perceptual electrode discrimination (Ramos de Miguel et al., 2018). The results comparing speech perception outcomes between array types are mixed, with some studies showing better performance with the precurved arrays on both pure-tone audiometry (PTA) and consonant-nucleus-consonant (CNC) tests (Holder et al., 2019), and others showing no difference with the same tests (Garaycochea et al., 2020). Although one might expect differences in electrode location and in degrees of channel interaction between electrode array types based on their design, the authors are unaware of a high-powered, publically-available report that concretely demonstrates the intended differences between them and how this may vary at different locations within the cochlea. This highlights the value in investigating and quantifying potential array-type differences using a detailed method.

The Panoramic ECAP Algorithm (PECAP) is a method that uses a myriad of Electrically Evoked Compound Action Potentials (ECAPs) from along the entire electrode array of a cochlear implant user and infers the underlying neural excitation patterns (Garcia et al., 2021). Using the forward-masking artefact-reduction technique, ECAPs are recorded for every combination of masker and probe electrode at the most comfortable listening level (MCL) for each electrode. Based on the assumption that every ECAP amplitude reflects the joint neural activation of both the masker and probe electrodes’ activation patterns, it treats this as a deconvolution problem and estimates the relative contribution of the neural responsiveness and the current spread at each electrode to these activation patterns. As noted by Garcia *et al* (2021) and Bieshuevel *et al* (2016), this approach is preferable to previous methods of estimating the width of the spread-of excitation (SOE) function where the probe is fixed on one electrode and the masker is moved to all other electrodes (Cohen et al., 2003), as it doesn’t assume infinitely-narrow masker electrode excitation patterns. Since it is based on ECAPs, the ‘neural-responsiveness’ estimate should not be construed as a general ‘neural-health’ estimate nor directly indicative of spiral ganglion nerve (SGN) survival. Rather, it is an indication of the relative synchronous neural responsiveness of neural tissue to electrical stimulation at a comfortable level compared to other locations along the array within an individual CI user’s cochlea (albeit at lower pulse rates and higher current levels than used in a typical CI patient’s MAP). It has been validated by selectively putting neurons near one electrode into a refractory state during PECAP data collection and observing a local reduction in PECAP’s estimate of neural responsiveness (Garcia et al., 2021). The current-spread estimate is modelled with symmetric Gaussian curves centered at each electrode, and has been validated using current-manipulation techniques that aim either to increase or decrease the current spread achieved whilst stimulating in standard monopolar (MP) mode (Garcia et al., 2024). PECAP therefore provides a tool to investigate how the synchronous responsiveness of the auditory nerve and the spread of electrical current vary along the length of the electrode array for an individual CI user. An online implementation of PECAP is also available, providing users with tools both for collecting and analyzing new data (Garcia & Lazauskas, 2024). A potential clinical application could arise by identifying “unusual” patterns – such as localized reductions in neural responsiveness or wide current spread – in a patient who gains little benefit from their CI. More generally, it can also provide a platform with which to compare current spread at different locations within the cochlea between different electrode array types. Both of these applications would benefit from a normative data set describing PECAP’s current-spread and neural-responsiveness measures in a larger number of implanted ears.

Through various international collaborations, a substantial dataset of PECAP measurements from 91 users of cochlear implant devices from Cochlear (Sydney, Australia) was available. This dataset included variation in electrode array type that covered all three designs manufactured by Cochlear: “Slim Straight” electrode arrays that are designed to be placed along the lateral wall of the cochlea (CI-22 series), “Contour Advance” electrode arrays that are precurved and designed to place its electrodes closer to the nerve (CI-12 series), and “Slim Modiolar” arrays that are a slimmer version of the Contour Advanced arrays (CI-32 series). Estimates of electrode-modiolus distances (EMDs) derived from Computed tomography (CT) scans were also available for 41 of the 91 ears.

A second dataset of PECAP measurements from 53 users of cochlear implant devices from Advanced Bionics (Valencia, California, USA) was also available. This dataset also included variation in electrode array type: “1J” and “SlimJ” are straight arrays designed to protect cochlear structures, whereas “Helix” and “Mid-Scala” are precurved arrays designed for ‘consistent positioning in the scala tympani to protect the delicate cochlear structures (Hassepass et al., 2014; *Internal Components of a Cochlear Implant* | *Advanced Bionics*, n.d.).’ No CT scans were available for this second dataset.

Post-hoc analyses were conducted to investigate whether PECAP revealed any differences in current spread and/or relative neural responsiveness between different electrode array designs and/or for different electrodes within the cochlea for both manufacturers. We note that, whereas PECAP’s estimate of current spread is expressed in absolute terms and allows direct comparisons both of the average current spread and its variation along the array between CI users, the neural-responsiveness estimate is a normalized measure that only reflects the relative pattern of responsiveness along the cochlea for a given participant (Garcia et al., 2021). Therefore, any across-participant differences in average neural responsiveness estimated by PECAP cannot, by definition, describe absolute differences in overall neural-responsiveness between CI users. Any across-participant assessments of the absolute values of this estimate are therefore primarily useful in serving as a sort of negative control. We *were*, however, able to examine the effect of the interaction between cochlear location and array type on the *(relative)* neural responsiveness estimate, as this preserves the within-participant, across-electrode variation the estimate is designed to capture.

We also adopted a metric (Peng et al., 2023) that captures the variation in the neural-responsiveness estimate between adjacent electrodes, and that has been shown to correlate with variations in another proposed estimate of neural responsiveness, namely the difference between detection thresholds with monopolar vs bipolar stimulation (Goldwyn et al., 2010). In our implementation we first calculate the difference between the neural responsiveness estimate between adjacent electrodes and then the variance in this difference measure along the array.

## Materials and Methods

### Cochlear

Panoramic ECAP Data was collected from 91 ears of 90 users of Cochlear CI devices at five different research centers. For Center 1, custom software developed using the Nucleus Implant Communicator (NIC2) platform provided by Cochlear (Sydney, Australia) was used to collect ECAP data for every combination of masker and probe electrode using the forward-masking artefact-reduction technique at most comfortable level (MCL). Ethical approval to conduct the study was granted in accordance with the International Research Code of Ethics (1990). Each additional center was provided with instructions and MATLAB code necessary to collect the same ECAP data as described above using Custom Sound EP (Sydney, Australia). These resources are available in the GitHub repository associated with this article. Centers 3 and 4 recorded an abbreviated set of data compared to the other centers, wherein ECAPs were only recorded for the conditions where the masker was presented on the same or more-basal electrodes relative to the probe electrode. This saved time in data-collection sessions and it has been shown previously that this dataset is sufficient to estimate neural responsiveness and current spread using the PECAP method (Garcia et al., 2023). Centers 2-5 obtained their own ethical approval for collecting their own data. All PECAP datasets included achieved the 10 dB SNR threshold identified by Garcia *et al*. (2021) for reproducing known underlying neural excitation patterns with at least 90% accuracy. Details of the array types and centers at which the data were collected can be found in Table 1.

**Figure 1:**
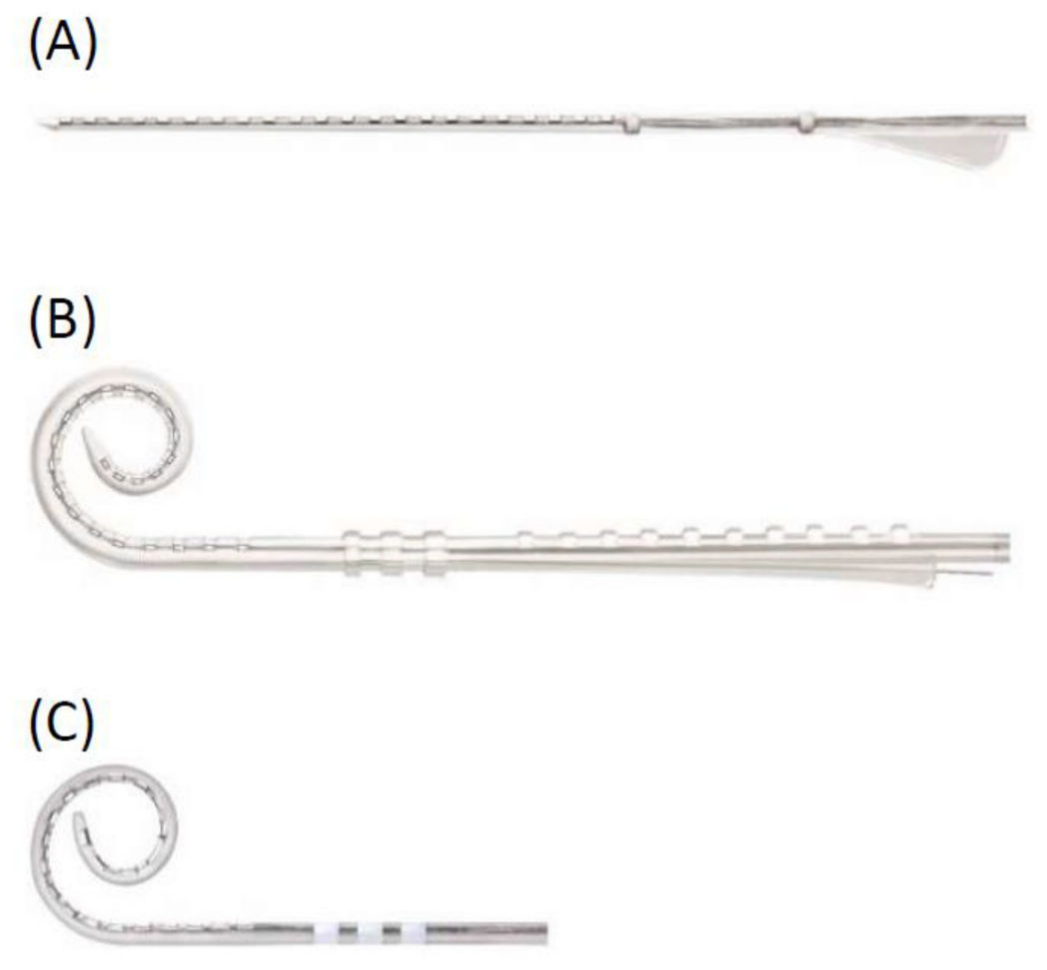
Cochlear © electrode array designs. The Slim Straight (A) is the ‘thinnest full length electrode” array. It has a “soft top combined with thin diameter, apical flexibility, and smooth lateral wall surface [that] facilitates an easy single stroke insertion designed to protect the delicate cochlear structures.” The Contour Advance (B) is a “stylet based perimodiolar electrode” array that “gets closer to the nerve through its unique design.” The Slim Modiolar electrode (C) is a thin full-length perimodiolar electrode “designed for consistent scala tympani placement” and “combines the unique benefits of a thin electrode with the perimodiolar positioning” close to the nerves. All descriptions and images provided by Cochlear © (*Nucleus Implant Information Center*, n.d.) (n.d. = no date)

The total Cochlear sample contained PECAP data from 35 Slim Straight (CI-22) arrays, 43 Contour Advance (CI-12) arrays, and 13 Slim Modiolar (CI-32) arrays. Descriptions and graphics representing each of these three array types can be found in Figure 1. Each PECAP dataset contained ECAP waveforms for each combination of masker and probe electrodes using the forward-masking artefact reduction technique, with the recording electrode placed 2 electrodes apical to the probe by default. When the probe was located on either of the two most apical electrodes, the recording electrode was placed 2 electrodes basal to the probe. In all conditions, if the recording electrode would have by default been placed on the same electrode as the masker, the recording electrode was instead placed between the masker and the probe to avoid stimulating and recording on the same electrode. All stimulation was with cathodic-leading biphasic current pulses presented in monopolar (MP) mode with 400 µs masker-probe intervals. A variety of phase-duration, gain, and delay settings were used for different CI users to obtain the clearest ECAP waveforms possible, selected from 25, 37, 42 or 50 µs, 40, 50, or 60 dB and 73, 98, and 122 µs, respectively. These parameters remained fixed across electrodes within each individual participant and did not vary systematically between array geometries; 82 of the 91 ears used the shortest phase duration of 25 μs, and five of the Slim Straight arrays and four Contour Advance arrays used longer phase durations. The default and recommended settings are a phase duration of 25 μs, a Gain of 50 dB, and a Delay of 98 μs. Each site was instructed to increase the phase duration if MCL could not be reached within compliance, and vary the other parameters as necessary to minimize N1 peak clipping and amplifier saturation. ECAP Amplitudes were calculated using custom peak-picking software to determine the latency and voltage of the negative (N1) and positive (P2) peaks. Waveforms were inspected visually to confirm expected ECAP waveform morphology of a N1 peak followed by a broader P2 peak, and failing this, to identify and exclude recordings that contained electrical artefacts (such as large, rapid voltage changes) that obscured neural responses. The amplitudes were assembled into NxN matrices for each CI user (referred to as *M*_0_), where N represents the number of electrodes active in that user’s MAP (up to 22). ECAPs that contained electrical artefacts were removed from the matrices and replaced with the amplitude of the opposite corresponding masker-probe combination (e.g. if there was an electrical artefact when the masker was on electrode 2 and the probe on electrode 5, then this cell in the matrix was replaced with the ECAP amplitude calculated when the probe was on electrode 2 and the masker on electrode 5). This method was considered valid due to evidence that test-retest reliability metrics of PECAP *M*_0_ matrices are statistically indistinguishable from those of symmetric PECAP *M*_0_ matrices constructed with each cell equal to the average of *M*_(*p*,*m*)_ and *M*_(*m*,*p*)_ where *M*_(*i*,*j*)_ represents the ECAP amplitude when the probe is presented on electrode *i* and the masker is presented on electrode *j* (Garcia et al., 2023). If this was unavailable because the artefact was present in a waveform where the masker and probe were co-located, the opposite corresponding masker-probe combination also contained an electrical artefact, or only half the *M*_0_ was recorded as was the case for data from Centers 2 and 3, then these values were linearly interpolated from adjacent ECAP-amplitude cells within the matrix. These substitutions or interpolations were only performed for 0.56% of ECAPs in this dataset (0.16% of all ECAPs from Slim Straight arrays, 0.99% from Contour Advance arrays, and 0.24% from Slim Modiolar arrays). Once complete, all PECAP *M*_0_ matrices were submitted to the Panoramic ECAP Algorithm as described by Garcia, *et al* (2021) to extract estimates of neural responsiveness and of current spread for each electrode switched on in each CI participant’s MAP.

For 41 of the participants from Centers 3 and 4 referred to in Table 1, Computed Tomography (CT) scans were also available, whereas this data was not collected for the other centers. Electrode-Modiolus Distances (EMDs) were calculated from these scans by those centers using a semi-automated software developed by the European Institute for Otolaryngology (EIORL, Antwerp, Belgium) that measures the distance between the center of the electrode and the medial wall in a 3-D rendering of the cochlea based on the CT imaging (Sismono et al., 2022). Linear Mixed Effects Models were fit to determine whether PECAP estimates and EMDs varied by electrode and/or by array type, as well as whether the EMD and PECAP’s estimate of current spread correlated with each other. As all analyses are post-hoc and not pre-planned prior to data collection, the statistical tests are described in the results section.

### Advanced Bionics

Panoramic ECAP Data was also collected from 53 ears of 52 users of Advanced Bionics CI devices at five different research centers. Data was collected using custom software developed in MATLAB (Natick, Massachusetts, USA) version 2018a in connection with the Bionic Ear Data Collection System (BEDCS) platform provided by Advanced Bionics (Valencia, California), version 1.18. This software performed loudness scaling to determine MCL for different electrodes and then recorded ECAP data for every combination of masker and probe electrode using the forward-masking artefact-reduction technique, similar to the procedures described above for the Cochlear platform. For Center 1, ethical approval to conduct the study was granted from Health and Care Research Wales (HCRW) through University Hospitals NHS Foundations Trust and the University of Cambridge (IRAS: 285894, Ref no: A095798, REC Reference: 20/EM/0263). Each additional center was provided with instructions and the same MATLAB program to collect PECAP data. These resources are available in the GitHub repository associated with this article. Each additional center obtained their own ethical approval for collecting their own data. As with the Cochlear data, these PECAP datasets included achieved the 10 dB SNR threshold identified by Garcia *et al*. (2021) for reproducing known underlying neural excitation patterns with at least 90% accuracy. Details of the array types and centers from which the data came from can be found in Table 2.

**Figure 2:**
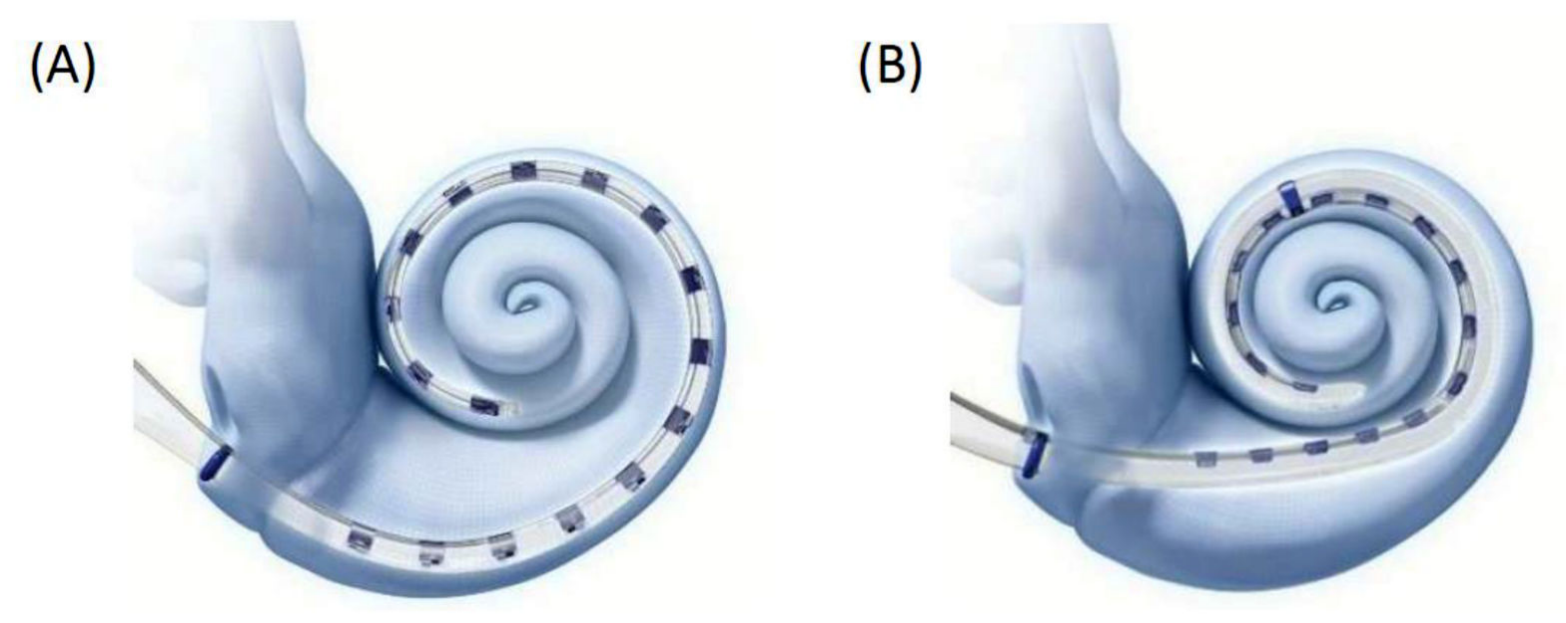
Advanced Bionics © electrode array designs. The HiFocus SlimJ (A) is a “straight electrode with a gentle curvature, designed to be easily and smoothly inserted by freehand technique.” This is the newer, slimmer model of the older straight ‘1J’ model, with similar specifications. The HiFocus Mid-Scala (B) “is the smallest styleted precurved electrode designed for consistent positioning in the scala tympani to protect the delicate cochlear structures.” It is the newer model of the older ‘Helix’ precurved array that was designed as a perimodiolar array, whereas the mid-scala array, as the name suggests, is designed to sit in the middle of the scala tympani. All descriptions and images provided by Advanced Bionics © (*Internal Components of a Cochlear Implant* | *Advanced Bionics*, n.d.)

The total Advanced Bionics sample contained PECAP data from 9 1J arrays, 20 SlimJ arrays, 4 Helix arrays, and 20 Mid-Scala arrays. Descriptions and graphics representing the newer two of the four array types can be found in Figure 2. Differences in ECAP recording parameters from the Cochlear platform included no delay parameter (the entire electrical artefact was also recorded), a standard gain of 1000, masker-probe intervals (MPI) of 500-600 µs, and phase durations of 22, 32, 43, or 54 µs. The default and recommended settings were a 500 μs MPI and a phase duration of 43 μs. Centers 2 and 4 used MPIs of 600 μs to avoid electrical artefacts resulting from amplifier saturation. Center 1 varied the phase duration to match each patient’s clinical settings as close as possible, and Center 2 used phase durations of 32 μs. There were no systematic differences in phase duration between array types; 37 of the 53 ears used the default 43 μs, 13 used 32 μs (7 1Js, 2 SlimJs, 3 Mid-Scalas, and 1 Helix), 2 used 22 μs (1 SlimJ and 1 Mid-Scala), and 1 used 54 μs (a Mid-Scala). The PECAP matrices were assembled using the same procedure for peak-picking and artefact removal as described in the previous section for Cochlear devices. Substitutions or interpolations were only performed for 0.75% of ECAPs in this dataset (0.8% of all ECAPs from 1J arrays, 1.21% from SlimJ arrays, 0.42 from Mid-Scala arrays, and 0.1% from Helix arrays). These *M*_0_ matrices were then submitted to the PECAP algorithm to extract estimates of neural responsiveness and current spread for each electrode for each cochlear implant user. The only CT scans available were for those at Center 2, and as these data have already been compared to PECAP estimates in Garcia, *et al*. (2021), no EMD measurements were assessed as part of this investigation for Advanced Bionics devices. LME Models were also fit for the Advanced Bionics dataset to determine whether PECAP estimates varied by electrode and/or by array type. As all analyses are post-hoc and not pre-planned prior to data collection, the statistical tests are described in the results section.

## Results

### Cochlear

**Figure 3:**
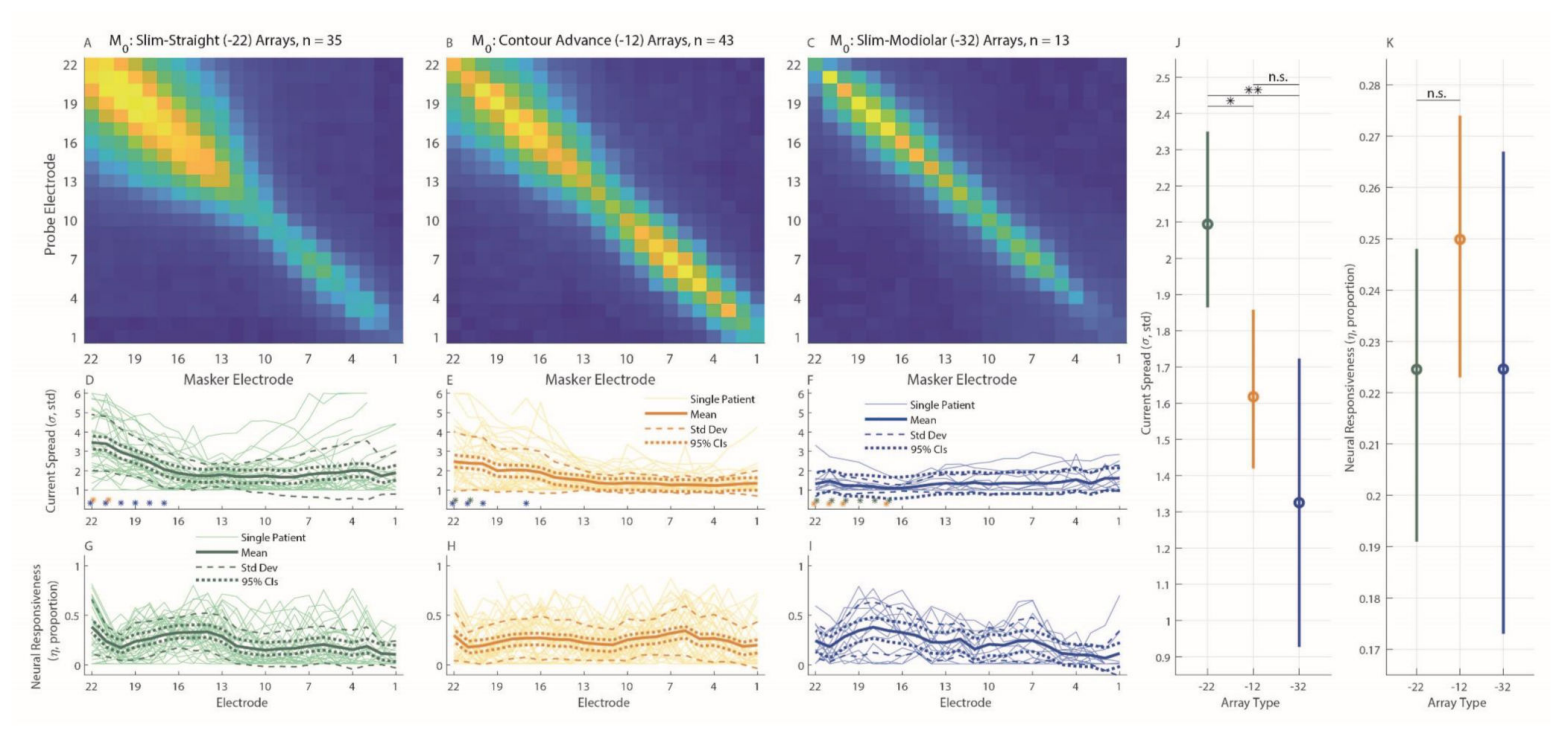
PECAP’s measurement matrix data, normalized and averaged across ears for each array type (A-C, with values ranging between 0 and 1), with PECAP’s current-spread (σ) estimates (D-F) and neural-responsiveness (η) estimates (G-I) included below. Results for the Slim Straight (CI-22) arrays are represented in green (A,D,G), Contour Advance (CI-12) in orange (B,E,H), and Slim Modiolar (CI-32) in blue (C,F,I). In the current-spread and neural-responsiveness panes (D-I), the thick solid lines represent the group-level mean, the thin solid lines represent individual CI user ears, the dashed lines represent one standard deviation from the group-level mean, and the dotted lines represent 95% confidence intervals. The asterisks in panes (D-F) represent the post-hoc comparisons of the interaction on the current-spread estimate of array type and electrode, the location representing the electrode and the colour representing the array type. The tall left panel (J) shows PECAP’s current-spread estimate by array type, and the tall right panel (K) shows PECAP’s relative neural-responsiveness estimate by array type. Error bars represent 95% confidence intervals. * *p* < 0.05, ** *p* < 0.01

The top plot in each column of Figure 3 (Fig. 3A-C) shows the measurement matrix for each array type, averaged across participants. Exemplary ECAP waveform data is not shown here to avoid misrepresenting the overall reliability of the data. The middle and bottom plots show PECAP’s current-spread (σ) and neural-responsiveness (η) estimates, respectively (Fig. 3D-F; Fig. 3G-I), with mean data shown by the bold solid lines, the mean ± one standard deviation shown by the dashed lines, and the 95% confidence intervals shown by the dotted lines. A Linear Mixed Effects (LME) Model was fit in Statistical Package for Social Sciences (SPSS) Software version 28.0.0.0 (IBM, Armonk, New York, USA) with PECAP’s current-spread estimate (σ) as the dependent variable, Array Type (3 levels) and Electrode (22 levels) as fixed factors, and Center as a random factor. An intercept and an interaction term of the two fixed factors were also fit, and Electrode was specified as a repeated variable to which a correction for sphericity was applied via a first-order autoregressive process. The LME found significant main effects of both array type (F(2,94.64) = 6.99, *p* = 0.001) and electrode (F(21,1405.83) = 3.73, *p* < 0.001), as well as a significant interaction (Array Type x Electrode) between them (F(42,1407.41) = 1.65, *p* = 0.006). After applying corrections for multiple (3) comparisons via the Bonferroni-Dunn method, pairwise comparisons computed following the LME found that Slim Straight (-22) arrays showed higher current spread than both the Contour Advance (-12) and Slim Modiolar (-32) arrays (*p* = 0.016 and 0.004, respectively), but that the two precurved arrays were not statistically significant form each other (*p* = 0.519). Pairwise comparisons (corrected for comparisons of 22 levels) also revealed that apical electrodes (electrodes 20-22) showed higher current spread than the majority of the rest of the array (electrodes 3-18). The significant interaction showed that for the two apical-most electrodes (electrodes 22 and 21), the current spread was wider for the Slim Straight (-22) arrays (95% CIs, e21: 3.05 – 3.73 σ, e22: 3.12 – 3.79 σ) than for the Contour Advance (-12) (95% CIs, e21: 2.18 – 2.79 σ, e22: 2.24 – 2.86 σ) which in turn was wider than for the Slim Modiolar (-32) (95% CIs, e21: 0.92 – 2.02 σ, e22: 0.77 – 1.87 σ) ones. Slim Straight (-22) arrays also showed wider current spread for electrodes 17 through 20 (95% CIs, e17: 1.73 – 2.40 σ, e18: 2.10 – 2.77 σ, e19: 2.38 – 3.05 σ, e20: 2.63 – 3.30 σ) than for Slim Modiolar (-32) arrays (95% CIs, e17: 0.54 – 1.64 σ, e18: 0.62 – 1.72 σ, e19: 0.67 – 1.78 σ, e20: 0.68 – 1.78 σ). Contour Advance (-12) arrays only additionally showed wider current spread for electrodes 17 and 20 (95% CIs, e17: 1.72 – 2.33 σ, e20: 2.09 – 2.70 σ) than for the Slim Modiolar (-32) arrays (95% CIs, e17: 0.54 – 1.64 σ, e20: 0.68 – 1.78 σ). An additional LME Model wherein the Slim-Straight arrays were removed still showed a significant interaction of Array Type x Electrode (F(21,812.10) = 1.74, *p* = 0.022), suggesting that the patterns of current spread do differ between the two precurved arrays more generally. Note that all *p*-values associated with significant factors, interactions, and pairwise comparisons have been corrected for multiple comparisons by multiplying the *p*-value directly by the number of comparisons made, to maintain a significance threshold of α = 0.05.

The same LME Model (with all three array types included) was computed for PECAP’s relative neural-responsiveness estimate (η) and found a significant main effect of Electrode (F(21,1119.61) = 4.20, *p* < 0.001) but no significant effect of Array Type (F(2, 208.26) = 1.27, *p* = 0.283, nor a significant interaction (F(42,1138.75) = 1.40, *p* = 0.58). The effect of Electrode showed a slight sloping trend across electrodes with 7.4 – 27.2% higher values at the apex than the base, with electrodes 14-19 and 22 all significantly greater neural-responsiveness values than electrodes 1 & 2 (*p-*values ranging from 0.050 to < 0.001), and electrodes 7 & 6 also showing greater values than for electrode 2 (*p-*values = 0.015 for both comparisons). As noted in the Introduction, the neural-responsiveness estimate is normalized (driven by the size of the highest ECAP amplitude in the measured *M*_0_ for each participant) so as to reveal only the *relative* change in responsiveness along the cochlea, and so no main effects of array type would be expected, by definition. Individual and summary PECAP results are depicted in Figure 3 (Fig. 3D-I), as well as means and 95% confidence intervals by array type for both the current-spread and neural-responsiveness estimates (Fig. 3J-K).

As ECAPs that constitute PECAP’s *M*_0_ matrices are recorded at MCL, it might be expected that any effects observed on the algorithm’s outputs may be influenced by the current level required to achieve MCL. As such, the same LME Model (with all three array types included) was again computed with current required to achieve MCL as the dependent variable. The LME found a significant main effect of electrode (F(21,1503.15) = 1.93, *p* = 0.007, but no significant effect of array type (F(2,112.381) = 1.425, *p* = 0.245) nor the interaction (Array Type x Electrode) between them (F(42,1504.90) = 0.81, *p* = 0.797). Pairwise comparisons (corrected for comparisons of 22 levels) revealed that central electrodes (electrodes 10-14) used higher MCLs than apical electrodes (electrodes 20-22).

The across-electrode variation in PECAP’s neural-responsiveness estimate is of more interest than its normalized absolute value. To better characterize this variation, we calculated the metric described by Peng *et al* (2023) that was designed to identify the presence of local “dead regions” whilst being unaffected by gradual changes in responsiveness along the array. To do so it computes the standard deviation of the differences between the log of the neural-responsiveness estimates for adjacent electrodes (Peng et al., 2023). A one-way ANOVA with this neural-responsiveness variation metric as the dependent variable and Array Type (3 levels) as the factor was computed in MATLAB 2020b (Mathworks, Natick, Massachusetts, USA) and showed no effect of Array Type (F(2,88) = 1.86, *p* = 0.1616). The across-group mean of the neural-response variation metric was 0.31 – 1.99 dB (95% confidence intervals). This analysis therefore found no evidence for between-array differences in the size of local variations in neural responsiveness along the cochlea.

**Figure 4:**
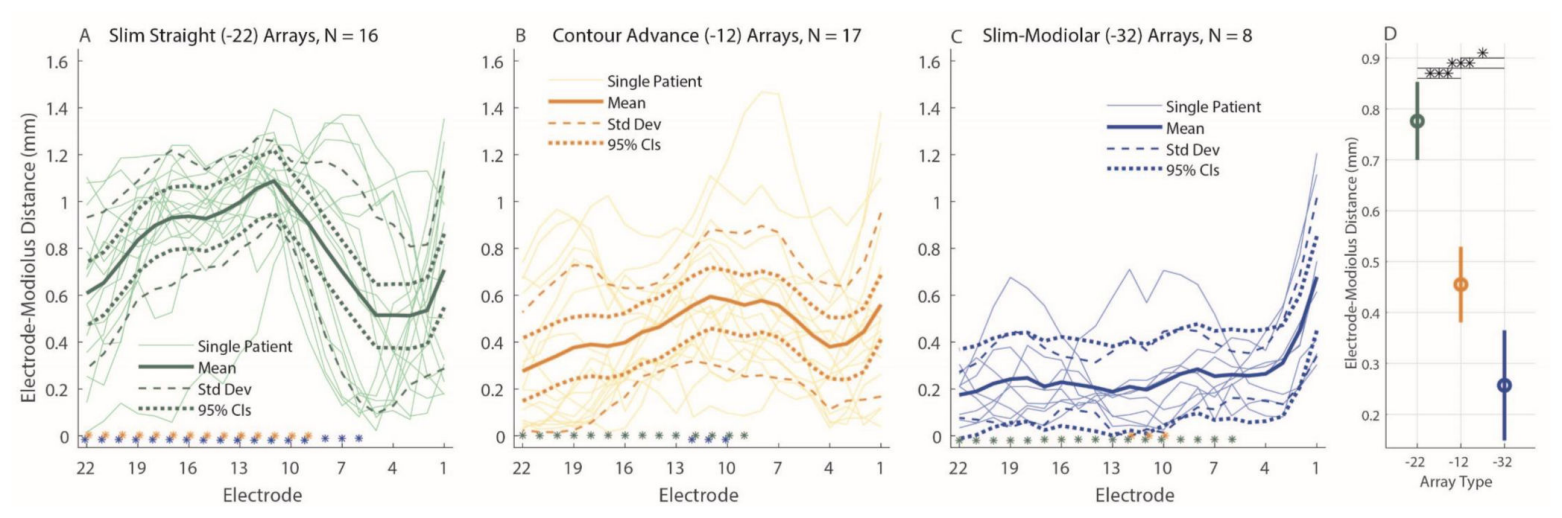
Electrode-Modiolus Distances (EMDs) by electrode for each Cochlear array type. Data from Slim Straight arrays are represented in green (A), Contour Advance in orange (B), and Slim Modiolar in blue (C). Thick solid lines represent group-levels means, thin solid lines represent individual CI user ears, dashed lines represent one standard deviation from the group-level mean, and dotted lines represent 95% confidence intervals. The asterisks in panes (A-C) represent the post-hoc comparisons of the interaction on the current-spread estimate of array type and electrode, the location representing the electrode and the colour representing the array type. The right-most panel shows the EMD by array type (D), with error bars representing 95% confidence intervals. * *p* < 0.05, *** *p* < 0.001

For 41 participants, CT scans were available and the electrode-modiolus distances (EMDs) are shown in Figure 4. This was the case for 16 Slim Straight arrays, 17 Contour Advance arrays, and 8 Slim Modiolar arrays. For this subset of data, an additional LME model was fit with EMD as the dependent variable, Array Type (3 levels) and Electrode (22 levels) as fixed factors, and Center as a random factor. As before, an intercept and an interaction term of the two fixed factors were also fit, and Electrode was specified as a repeated variable to which a correction for sphericity was applied via a first-order autoregressive process. This LME showed a significant main effect of Electrode (F(21,550.97) = 1.72, *p* = 0.025) and of Array Type (F(2,66.16) = 35.58, *p* < 0.001), and a significant interaction (Array Type x Electrode) between the two (F(42,552.53) = 5.48, *p* < 0.001) on the EMD. Similar to the effect of array type on PECAP’s current-spread estimate (σ), pairwise comparisons following the LME found that the EMD for the Slim-Straight arrays (CI-22) was higher than both the Contour Advance (CI-12, *p* < 0.001) and Slim Modiolar arrays (CI-32, *p* < 0.001) after applying corrections for multiple comparisons via the Bonferroni-Dunn method. In contrast to the effect of array type on PECAP’s current-spread estimate, the Contour Advance arrays also showed higher EMDs than the Slim Modiolar ones (*p* = 0.011). The main effect of electrode seemed to show a different pattern than the PECAP current-spread estimate; pairwise comparisons demonstrated that a few middle electrodes (e9-12) showed significantly larger EMDs than the most apical electrode 22 (e9: *p* = 0.036, e10: *p* = 0.026, e11: *p* = 0.005, e12: *p* = 0.014), and that electrode 11 also showed larger EMDs than electrodes 4 and 21 (e4: *p* = 0.048, e21: *p* = 0.005). The significant interaction demonstrated higher EMDs for apical and middle electrodes in Slim Straight arrays than when compared to the Contour Advance arrays (all electrodes from 9 - 22) and the Slim Modiolar arrays (all electrodes from 6 - 22). The EMDs were significantly smaller for the Slim Modiolar arrays than for the Contour Advance arrays for a few middle electrodes (all electrodes from 10 - 12). An additional LME model wherein the Slim Straight arrays were removed still showed a significant interaction of Electrode x Array Type (F(21,483.06) = 2.28, *p* = 0.001), suggesting that the patterns of EMDs do differ between the two precurved arrays more generally. Figure 4 shows the EMDs by electrode for each of the three array types (Fig. 4A-C) and the main effect of array type (Fig. 4D).

It appeared that the significant interaction between the array type and the electrode, while significant for both PECAP’s estimate of current spread and for the EMD measurements, were different in nature and affected different electrodes. For PECAP’s current-spread estimate, the interaction was largest at the most-apical electrodes, whereas for the EMD measurements the interaction was largest for central electrodes. An additional LME was conducted to confirm whether the interactions were statistically different from each other with Value as the dependent variable (either EMD or σ, normalized to unit variance to avoid unequal weighting between the measurements) and Measurement (2 levels, EMD or σ), Array Type (3 levels, -22, -12, or -32), and Electrode (22 levels) as fixed factors. A correction for sphericity via an autoregressive process could not be conducted because electrode could not be specified as a repeated measure due to repeat cases of each electrode per subject, so a manual correction for sphericity was performed using the lower-bound method. A significant 3-way interaction (Measurement x Array Type x Electrode) was found (F(42,75.71) = 2.95, *p* < 0.001), suggesting that the interaction between electrode position and array type did indeed differ significantly between the current-spread and EMD measures.

We also repeated the LME described above for PECAP’s current-spread (σ) and neural-responsiveness (η) estimates to determine whether the limited sample size (N = 41) still showed the array-type and electrode effects seen in the full sample. This showed the same main effects of Electrode (F = (21,538.25) = 9.70, *p* < 0.001) and Array Type (F(2,81.20) = 13.47, *p* < 0.001) as well as interaction (Electrode x Array Type, F(42,540.24) = 4.27, *p* < 0.001) on the current-spread estimate (σ), and the same main effect of Electrode (F(21,479.93) = 4.06, *p* < 0.001), a lack of effect of Array Type (F(2,110.31) = 1.00, *p* = 0.370) on the neural-responsiveness estimate (η), and a lack of interaction (F(42,491.01) = 1.14, *p* = 0.264). These results suggest that the subset of data (N=41) is representative of the larger sample size (N=91).

**Figure 5:**
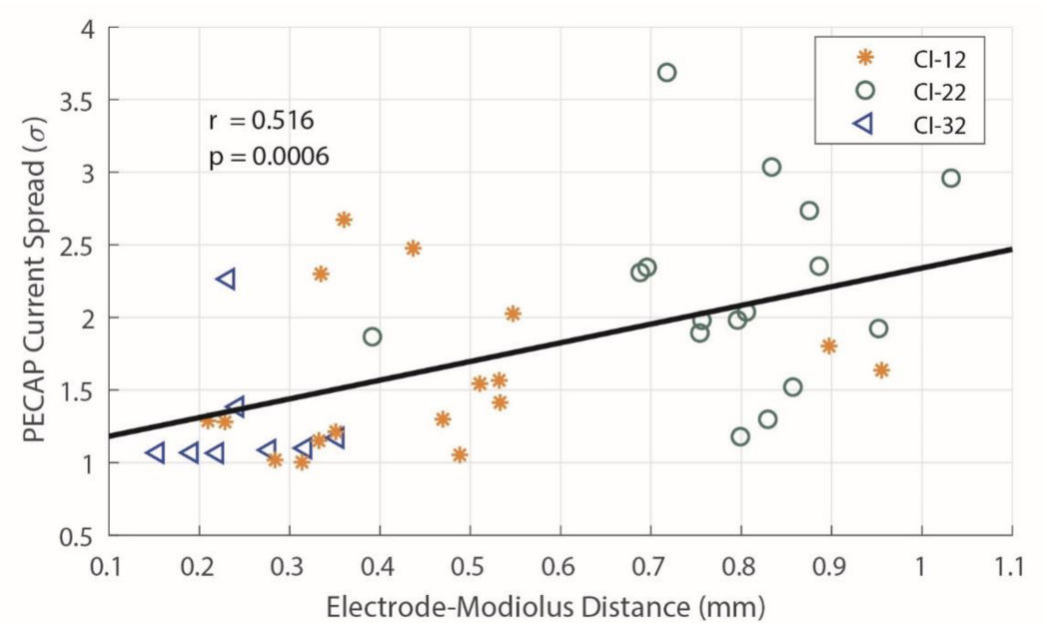
The across-participant correlation between the average EMD and average current-spread estimate (σ) for the 41 participants for whom both PECAP and CT scan data were available. Each symbol represents one CI ear, with the green circles representing users of Slim Straight (CI-22) arrays, the orange stars representing Contour Advance (CI-12) arrays, and the blue triangles representing Slim Modiolar (CI-32) arrays. The solid black line shows the positive correlation between the across-electrode means of both the EMD and current-spread estimate across participants

We also compared the PECAP’s current-spread estimates (σ) to the EMDs. Using MATLAB 2020b, we found a very small negative correlation across electrodes with between-participant differences removed (r(860) = -0.097, *p* = 0.004), but found a stronger positive correlation between across-electrode means of PECAP’s estimate of current spread (σ) and EMD across participants (r(40) = 0.516, *p* = 0.0006). This correlation, which is in the expected direction where greater current spread correspond to larger EMDs, is displayed in Figure 5.

### Advanced Bionics

**Figure 6:**
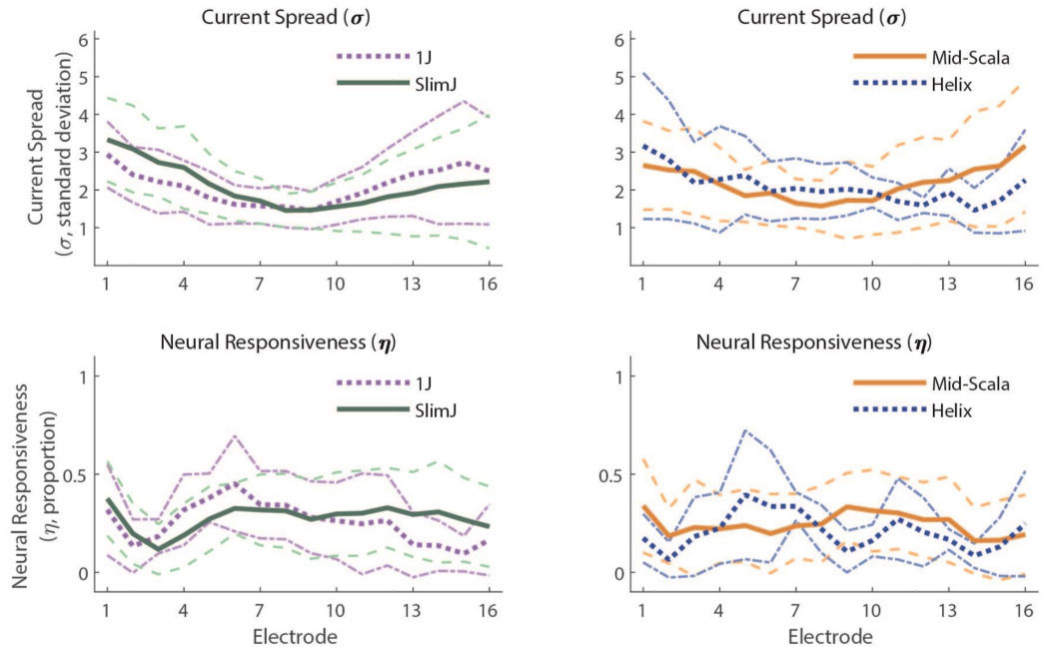
PECAP estimates of current spread (A,B) and neural responsiveness (C,D) for users of Advanced Bionics CI devices. Data for the straight arrays are displayed on the left (A,C) and for the precurved arrays on the right (B,D). For the older array-types (1J and Helix), thick dotted lines represent group-level means by array type, and thin dashed-dotted lines represent one standard deviation from the group-level mean. For the newer array types (SlimJ and Mid-Scala), thick solid lines represent group-level means by array type, and thin dashed lines represent one standard deviation from the group-level mean. Purple (dotted) lines represent data from 1J arrays, green (solid) from SlimJ arrays, orange (solid) lines from Mid-Scala arrays, and blue (dotted) lines from Helix arrays. No significant effect of array-type was found for the current-spread estimates, and an effect of electrode was found with the central electrodes (e5-12) showing lower current spread than apical electrodes (e1-2). (e = electrode)

PECAP data for the Advanced Bionics participants are shown in Figure 6. As with the Cochlear data, exemplary ECAP waveform data is not shown here to avoid misrepresenting the overall reliability of the data. An LME analysis was performed with PECAP’s current-spread estimate (σ) as the dependent variable, Array Type (4 levels) and Electrode (16 levels) as fixed factors, and Center as a random factor. An intercept and an interaction term of the two fixed factors were also fit, and Electrode was specified as a repeated variable to which a correction for sphericity was applied via a first-order autoregressive process. This LME showed a significant effect of Electrode (F(15,539.03) = 10.05, *p* < 0.001) on PECAP’s estimate of current spread, no significant effect of Array Type (F(3,80.81) = 0.063, *p* = 0.979), and a significant interaction (F(45,542.05) = 1.64, *p* = 0.007). After corrections for multiple comparisons via the Bonferroni-Dunn method, pairwise comparisons following the LME revealed that the middle electrodes (e5-12) showed narrower current spread than the two most apical electrodes 1 and 2 (compared to e1, e5: *p* = 0.007, e6-12: *p* < 0.001, e13: *p* = 0.001, e14: *p* = 0.007, and compared to e2, e5: *p* = 0.033, e6: *p* = 0.001, e7-9: *p* < 0.001, e10: *p* = 0.001, e11: *p* = 0.005, e12: *p* = 0.045), and electrode 8 additionally showed narrower current spread than electrodes 3 and 16 (e3: *p* = 0.028, e16: *p* = 0.027). The significant interaction showed that each of the significant pairwise comparisons described above was driven by differences in current-spread estimates by electrode in the SlimJ arrays (95% CIs for Slim J arrays, e1: 2.89 – 3.78 σ and e2: 2.64 – 3.53 σ > e5: 1.71 – 2.60 σ, e6: 1.39 – 2.28 σ, e7: 1.26 – 2.15 σ, e8: 1.01 – 1.90 σ, e9: 1.02 – 1.91 σ, e10: 1.11 – 2.00 σ, e11: 1.20 – 2.09 σ and e12: 1.43 – 2.32 σ), with the exception of e16 vs e8 which was driven only by differences in Mid-Scala arrays (95% CIs for Mid-Scala arrays, e16: 2.57 – 3.53 σ > e8: 1.13 – 2.02 σ). Lower current-spread estimates for central electrodes 7, 8, and 10 compared to apical electrodes 1 and 2 were additionally driven by differences in Mid-Scala arrays (95% CIs for Mid-Scala arrays, e7: 1.20 – 2.09 σ, e8: 1.13 – 2.02 σ and e10: 1.20 – 2.10 σ < e1: 2.15 – 3.06 σ and e2: 2.11 – 3.01 σ), and lower current-spread estimates for central electrodes 7-9 compared to apical electrode 1 were additionally driven by differences in 1J arrays (95% CIs for 1J arrays, e7: 0.91 – 2.24 σ, e8: 0.89 – 2.21 σ and e9: 0.80 – 2.13 σ < e1: 2.27 – 3.60 σ). These data are displayed in Figure 6 (Fig. 6A, 6B). The same LME was repeated for the neural-responsiveness estimate (η) and a main effect of Electrode (F(15,456.64) = 2.36, *p* = 0.003) was found, but no significant effect of Array Type (F(3,116.45) = 0.93, *p* = 0.427) nor interaction (F(45,460.10) = 1.17, *p* = 0.214). See Figure 6 (Fig. 6C, 6D). The effect of electrode was driven by highly variable differences showing higher neural-responsiveness for electrodes 5 and 6 than for electrodes 2 and 3 (≈ 0.1 – 33.6%, e2 vs e5: *p* = 0.005, e2 vs e6: *p* = 0.005, e3 vs e5: *p* = 0.025, e3 vs e6: *p* = 0.045).

The Peng *et al*. variation metric was also calculated to better characterize the neural-responsiveness estimate for the Advanced Bionics data. A one-way ANOVA with the neural-responsiveness variation metric as the dependent variable and Array Type (4 levels) as the factor showed no effect of Array Type (F(3,49) = 1.19, *p* = 0.3218). The across-group mean was 0.26 – 1.90 dB (95% confidence intervals).

## Discussion

The main effect of array type revealed by the analyses of both the PECAP and EMD data from Cochlear © devices presented here are generally consistent with each other and with expectations, showing the lowest current spread and electrode-modiolus distances for the precurved arrays (both Slim Modiolar and Contour Advance) and the highest for the Slim Straight arrays. The Contour Advance arrays sit between the two in terms of EMDs. On average, Slim Modiolar arrays showed EMDs between ≈0.15-0.37 millimeters (mm), Contour Advance showed EMDs between ≈0.38-0.53 mm, and Slim Straight showed EMDs between ≈0.70-0.85 mm (95% confidence intervals). The numerical value of PECAP’s current-spread estimates represents how many electrodes in each direction the current is estimated to spread from central electrodes. For Slim Modiolar arrays, this value was between ≈0.93-1.72 electrodes (≈0.56-1.03 mm) in each direction, for Contour Advance arrays it was ≈1.42-1.86 electrodes (≈0.85-1.12 mm), and for Slim Straight arrays it was ≈1.87-2.35 electrodes (≈1.68-2.12 mm) (each range describes 95% confidence intervals).

Some previous studies have shown results consistent with the effects of array type shown here: Xi *et al*. showed narrower spread of excitation for Contour CI24R(CS) compared to Straight CI24M arrays (Xi et al., 2009), Miller *et al*. showed greater channel interaction widths for the CI24M arrays than the CI24R arrays (as well as the CII implants that also incorporated some electrode curvature) (Miller et al., 2008) and Cohen *et al*. showed narrower forward masking profiles for Contour arrays compared to Straight ones, although this finding was not replicated in another study by the same group (Cohen et al., 2005, 2006). However, with the exception of Miller *et al* who include 39 ears, each of these studies included fewer than 15 participants, and to the authors’ knowledge the data presented here is the first investigation of current spread and spread of excitation using ECAPs between electrode array geometries with significantly larger sample sizes. Berg *et al*. (2024) conducted a review of spread of excitation in 238 CI users with both precurved and straight electrode array geometries, but they did not directly investigate any differences between the two.

Both the main effect of electrode number and, most importantly, its interaction with array type differed between the two measures investigated here. For the PECAP data, current spread was largest at the apex (electrodes 20 to 22) and the interactions suggest that the benefit of narrower current spread for the Slim Modiolar arrays over the Contour Advance and over the Slim Straight arrays in turn is also greatest at the most-apical electrodes, 21 and 22. Although the Slim Modiolar arrays did not have narrower current spread than the Contour Advance arrays overall, the interaction between electrode and array type did show an electrode-specific reduction in current spread between the two perimodiolar arrays for apical electrodes (e17 and e20-22). When interpreting these effects, however, it is important to note that they were computed for electrode number and uncontrolled for insertion depth, angle, or array length, for which the data were not available. If one expects current-spread variation to be due in part to variation in EMD, this particular effect is consistent with an experiment investigating ECAP thresholds and EMDs calculated from CT scans by Mewes *et al* where electrodes 20-22 showed higher EMDs for 19 Contour Advance (CI512) than for 27 Slim Modiolar (CI532) arrays (Mewes et al., 2020). However, and in contrast to Mewes *et al* who showed increasing EMDs for the basal half of the array compared to the apical half, EMDs were generally largest in the *middle* of the array between electrodes 9-12 for the present study, and the smaller EMD for the precurved arrays (again both the Slim Modiolar and Contour Advance) compared to the Slim Straight arrays is found both centrally and apically, from electrodes 9 to 22. The interaction *between* the two precurved arrays showed a narrower region of difference wherein the Contour Advance had wider current spread only for central electrodes 10-12 compared to the Slim Modiolar ones. The pattern of higher EMDs in the center of the array *is* consistent with a previous assessment of Cochlear’s perimodiolar arrays, observed in a study of 10 Contour Advance users before the development of the Slim Modiolar type (Long et al., 2014). Interestingly, Mewes *et al* also observed *higher* EMDs for the Slim Modiolar array than the Contour Advance array for electrodes in the basal half of the array (electrodes 2-10). While they did find a significant negative correlation between EMD and ECAP thresholds, they did not capture spread of excitation nor any ECAP measurements at MCL to compare to the PECAP findings here. A different investigation of 38 Cochlear users that did assess spread of excitation at 3 electrodes using ECAPs found wider spread of excitation (SOE) toward the base (electrode 5) than in the middle (electrode 13) or toward apex (electrode 18), although this assessment pooled all 3 array type geometries and leveraged an approach for estimating SOE from ECAPs that assumes uniform and infinitely narrow masker excitation profiles across the array (Kopsch et al., 2022). In contrast, a different assessment of 43 Slim Straight Cochlear arrays that leveraged the same SOE measure as Kopsch *et al* found narrower spread of excitation at the base than in the center or the apex of the array (Söderqvist et al., 2021). Another method that leverages a deconvolution technique similar to PECAP and that does not make this assumption has observed decreases in the width of excitation density profiles across the array from apex to base (Biesheuvel et al., 2016); both of these are consistent with the main effect of electrode shown in PECAP’s estimate of current spread here. Variation of observed effects in the literature underlie a need to further characterize both current spread and EMD along the electrode array for different array types in multiple cohorts, as well as a need to compare and/or align approaches for characterization of these aspects of the electrode-neuron interface.

The discrepancy observed here in the interactions between the two methods of assessing the electrode-neuron interface can be explained by the differences between the two measures. The EMD measurements are calculated from CT scans but include no consideration of the responsiveness of the nerves and how this may vary between CI users or between different places within the cochlea. It can be expected to somewhat affect the spread of electrical excitation from the electrodes to the neural tissue, but does not reflect other factors including fibrosis in the cochlea or the presence, orientation, and state of neurons in the auditory nerve. The PECAP estimate of current spread (σ), however, is estimated along the *length* of the cochlea (somewhat geometrically orthogonal to EMDs) and calculated using data reflecting the synchronized neural responsiveness of the tissue. While we can therefore expect the EMD to influence σ (especially in terms of how high the current must be in order to reach responsive neural tissue at different places along the cochlea), it may also be influenced by attributes of neural status along the cochlea such as neuron packing density and/or orientation. There is also variation in electrode spacing in the Cochlear arrays: Slim Modiolar electrodes have a uniform electrode spacing of 0.6 mm, but electrode spacing varies between 0.4 and 0.8 mm along the array for the Contour Advance electrodes and between 0.85 and 0.95 mm for the Slim Straight electrodes (*Nucleus Implant Information Center*, n.d.). The variation does not occur in a systematic apical-to-basal way (see supplemental information), so it cannot account for the effects we see on PECAP’s estimates of wider current spread at the apex than along the rest of the array. However, it might impact EMD and PECAP’s estimate of current spread differently: reduction in electrode spacing might cause changes in EMD to be smaller between adjacent electrodes, whereas it may give rise to an increase in PECAP’s estimate of current spread, because PECAP defines spread on an electrode scale and so a given spread in millimeters will correspond to a larger number of electrodes.

A significant across-participant correlation between the average EMD and average σ captures 27% of the variance between the two measurements, reflecting this relationship at the participant level, although there was a small (albeit significant) within-participant between-electrode correlation (describing <1% of the variance) in the opposite direction. The former across-participant correlation is consistent with findings of a 238-participant assessment of EMD and SOE widths by Berg *et al* in Slim Straight arrays, although the relationship is not observed in precurved arrays (Berg et al., 2024). It is also consistent with correlations observed between widths of Trans-Impedance Matrices and SOE curves (Söderqvist et al., 2021). The fact that even the between-participant correlation was modest (as are the correlations in other studies), combined with the different interactions between electrode and array-type for the current-spread and EMD measures, suggest that factors other than EMD dominate the current spread at the level of the neurons as estimated by PECAP.

Although we do not have a complete explanation for why the interaction between electrode number and array type differed between the two measures, it is worth noting a parallel between the effect of electrode position and of another manipulation designed to increase current spread, namely “blurring” the pattern of electrical stimulation by the simultaneous in-phase stimulation of multiple adjacent electrodes (Goehring et al., 2020). It has recently been reported that, compared to single-electrode monopolar stimulation, stimulating either 3 or 5 electrodes simultaneously increased PECAP’s estimate of current spread, but only did so when the blurring was applied to more-apical (as opposed to more-basal) electrodes (Garcia, et al., 2024). Both the blurring data and the data presented here are consistent with the idea that increasing current spread at the level of the cochlea is reflected in PECAP’s estimate of current spread only toward the apex. We cannot be sure that this actually corresponds to an increase in neural spread of excitation that occurs only at the apex without performing further psychophysical and/or modelling studies, but note that there is nothing inherent to the PECAP algorithm that treats the apical end of the array differently from the base. It may also be relevant that blurring apical (but not basal) electrodes affects speech perception (Goehring et al., 2021), although this latter finding could be due to the different amounts of speech information conveyed at the apex compared to the base. We also note that PECAP only captures *synchronized* neural activity from the cochlear nerve at low pulse rates. It is possible that non-synchronized neural activity as well as the behavior of neural tissue in response to higher pulse rates more typical of a clinical coding strategy contribute significantly to the spread of excitation experienced in every-day listening of cochlear implant users, and this would not be captured in PECAP’s estimate of current spread. For instance, Peng *et al* (2023) showed that their PECAP-derived measure of the variation in neural responsiveness along the cochlea correlated with variation in differences between monopolar and bipolar thresholds at high pulse rates, but only the across-electrode variation in the monopolar-bipolar differences correlated with performance on CNC phoneme scores (Peng et al., 2023). Further work is needed to investigate the relationship between PECAP’s estimates of neural excitation patterns and behavioral measures, and also in the development of methods that capture detailed neural excitation patterns at rates more similar to those used in clinical coding strategies.

The analysis of the Advanced Bionics PECAP data did not show a difference in current spread between different array types. This may be due to differences in electrode-array design when compared to Cochlear: mid-scala arrays are not designed to sit directly next to the cochlear nerve, but rather in the middle of the scala tympani, as the name suggests. The now-discontinued Helix array types were designed as perimodiolar but there were only 4 of these arrays included in the dataset presented here, limiting the ability of the analysis to see any potential differences. It’s possible that the lack of effect found reflects smaller differences between different array types in the Advanced Bionics platform than in the Cochlear platform. If there are any differences, the PECAP method is not sensitive enough to detect them with the statistical power afforded by the sample size used here. However, it’s also possible that the parameters used to collect the PECAP data for the AB device limited its sensitivity. The masker-probe method used here and elsewhere to measure ECAPs assumes that, when masker and probe are presented to the same electrode, the masker completely suppresses the response to the probe. Recent evidence suggests that the absolute refractory period of auditory nerve fibers may be shorter than even the 400 μs used as a standard Masker-Probe Interval (MPI) used in the forward masking artefact reduction paradigm for recording ECAPs in the Cochlear device (Skidmore et al., 2024), and so this would have been even more of an issue for the AB device where MPIs of 500 or 600 μs were used. Future iterations of the method – regardless of manufacturer – should consider using shorter MPIs, such as 350 μs or even 300 μs (Morsnowski et al., 2006). As there were no CT scans available for this dataset, it would also be beneficial for future work to investigate differences in EMD between array types for Advanced Bionics CIs.

While there were no differences in relative neural responsiveness found between array types in neither Cochlear nor Advanced Bionics devices, the PECAP method is not designed to assess differences in neural responsiveness *between* participants, but rather between electrodes *within* a CI user. Therefore, this result should *not* be taken as evidence that there are no differences in neural responsiveness between different array types from either manufacturer. However, as mentioned above, the PECAP-derived variation metric capturing differences in adjacent-electrode neural-responsiveness has been shown to correlate with the same metric describing variation of differences in monopolar vs bipolar thresholds. This latter metric is hypothesized to capture “off-site listening” potentially caused by local dead regions, and has been shown to correlate with performance on speech tests (Peng et al., 2023). We reasoned that calculating this variation metric for PECAP’s neural-responsiveness estimate might therefore capture some variation in neural health important for perception. As there were no differences in this metric found between array types for either the Cochlear or Advanced Bionics devices, the results presented here do not provide evidence of any potential trauma to the auditory nerve that would be reflected in variations in local survival and that might be caused by precurved electrode array designs. This is consistent with previous studies that fail to show a difference between straight and precurved arrays in hearing preservation (Jwair et al., 2021) or performances on sentence tests (Sharma et al., 2022), and others that fail to show correlations between speech scores and spread-of-excitation estimates that are narrower in precurved than in straight arrays (Berg et al., 2024). In fact, other studies have shown *higher* performance on word- and syllable-identification tasks for patients with precurved arrays compared to straight arrays (Sharma et al., 2022) and for those with narrower spread-of-excitation (da Silva et al., 2021), suggesting that benefits of narrower spread-of-excitation may outweigh potential deleterious effects of nerve damage in precurved arrays. While the results here do show the advantage to EMD and current spread of Cochlear’s precurved arrays, it should additionally be noted that the newer Slim Modiolar arrays show lower incidences of trans-location (≈5%) compared to previous Contour Advance generations (≈30%) (Liebscher et al., 2021), suggesting that some of the additional risks of trauma due to precurved electrode array design that may or may not be captured by PECAP may be mitigated by the Slim Modiolar design.

Although the data do seem to show an advantage of the precurved array types (especially the Slim Modiolar model) over the straight ones for Cochlear devices in terms of reducing spread of excitation and electrode-modiolus distances, we stress that all analyses presented here are post-hoc and therefore cannot confirm a causal link. Although a variety of cochlear implantation centers are included in the dataset, the authors have no information about the procedure for array type selection at each of those centers. One may reasonably expect that ENT surgeons select which array type to implant in a patient based on that particular patient’s etiology, as well as the surgeon’s experience with the insertion procedure for each, rather than selecting randomly. These factors could potentially have led to the differences in overall current spread between array types, and although it is harder to explain the interaction between array type and electrode position by these selection biases, this cannot be ruled out. Therefore, these analyses do not demonstrate that the selection of e.g. a Slim Modiolar array for any cochlear implant candidate would give rise to the excitation profiles and electrode-modiolus distances presented here. However, these patterns could be considered as part of the electrode array selection process, as they may characterize the degree to which electrical stimulation delivered to the cochlear nerve by the cochlear implant are optimized for auditory perception. Furthermore our results provide justification for a prospective trial of the effects of array type on the neural spread of excitation to investigate the possibility of a causal link.

**Figure 7:**
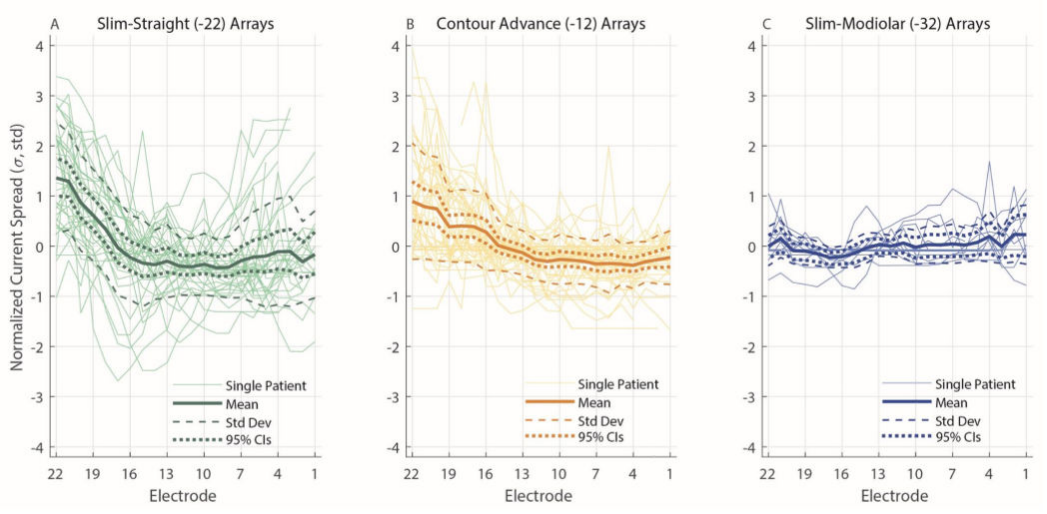
PECAP estimates of current spread, normalized by the average current-spread estimate across electrodes for each participant. Results for the Slim Straight (CI-22) arrays are represented in green (A), Contour Advance (CI-12) in orange (B), and Slim Modiolar (CI-32) in blue (C). Thick solid lines represent the group-level mean, the thin solid lines represent individual CI user ears, the dashed lines represent one standard deviation from the group-level mean, and the dotted lines represent the 95% confidence intervals.

Finally, in addition to providing evidence for the effect of array type and electrode position on current spread, our data provides a sizeable dataset that allows for characterization of the variation in PECAP’s current-spread and neural-responsiveness estimates. For the Cochlear device for which we have data from a total of 91 ears, the variation in current spread for a given electrode position appears greatest for the Slim Straight array and smallest for the Slim Modiolar array (see Figure 3). For example, for the most apical electrode, the estimates range from ≈ 2 – 5 σ (±1 standard deviation from the mean) for the Slim Straight array, but only ≈ 1 – 2 σ for the Slim-Modiolar array. The array-type difference in variation is broadly reflected in the electrode-modiolus distances as well (see Figure 4), and likely reflects the specifications of the different implanted arrays (*Nucleus Implant Information Center*, n.d.). However, since Figure 3 pools both across- and within-participant variation in current-spread estimates, we have additionally plotted these values relative to the mean value for each ear in Figure 7 to remove the between-participant source of variance. In practical terms, this may inform the clinician about the normative variation in current spread within a CI user for a particular array type, and could aid in identifying atypical cases. For this use case, it is the 95% confidence intervals displayed in Figure 7 that should be inspected, not the standard deviations. For instance, if the current spread for the apical-most electrodes was 1 σ above the mean for a Slim Straight or Contour Advance array, this would be typical, as a σ value of 1 falls within the 95% confidence intervals for the two most apical electrodes for both array types. However, the same variation in a Slim Modiolar array would not be typical, as the 95% confidence intervals for this array type do not include a value of σ above 1 for *any* electrode. However, the authors stress that the etiology of the patients included in this dataset is not known, and therefore all statistics reflect a *normative* description of PECAP data, but cannot be characterized as *normal*.

## Conclusion

The Panoramic ECAP Method reveals wider current spread for Slim Straight electrode arrays than for precurved ones (both Contour Advance and Slim Modiolar) developed by Cochlear ©, with the largest differences observed in the apex. CT scans in a subset of patients show largest electrode-modiolus distances for Slim Straight arrays followed by Contour Advance and then Slim Modiolar arrays, with smaller EMDs of the precurved arrays in the middle of the array as well as toward the apex. No differences in current spread as estimated by PECAP were observed in the Advanced Bionics devices. The results presented here provide a normative dataset against which to compare future PECAP data and may aid in identifying atypical cases. They also provide evidence of differences between array types that could be leveraged as part of the electrode array selection process in surgical practice, as they may impact the optimization of electrical stimulation delivered to the cochlear nerve by the cochlear implant. However, the authors underscore that all analyses presented here are post-hoc and therefore cannot represent a causal relationship between array type selection and patterns of current spread.

## Supplementary Material

Supplemental Information

## Figures and Tables

**Table 1:** Centre & Array Type Information for CI users of Cochlear devices

*Centre #*	*Centre*	*Slim Straight* *(CI-22)*	*Contour Advance* *(CI-12)*	*Slim Modiolar* *(CI-32)*
1	MRC Cognition & Brain Sciences Unit, University of Cambridge, UK	N = 9	N = 8	N = 1
2	Bionics Institute, Melbourne, Australia	N = 5	N = 6	N = 3
3	Hannover H Medical Centre, Germany (PREVA Study, Cochlear)	N = 10	N = 23	N = 5
4	Melbourne, Australia (PREVA Study, Cochlear)	N = 11	N = 5	N = 4
5	Boys Town National Research Hospital, Boystown, Nebraska, US	N = 0	N = 1	N = 0
Total	n/a	N = 35	N = 43	N = 13

MRC = Medical Research Council, N = number of ears

**Table 2:** Centre & Array Type Information for CI users of Advanced Bionics devices

*Centre #*	*Centre*	*Straight Arrays*		*Precurved Arrays*	
		*1J*	*SlimJ*	*Helix*	*Mid-Scala*
1	MRC Cognition & Brain Sciences Unit, University of Cambridge, UK	N = 1	N = 4	N = 0	N = 4
2	University of Washington, Seattle, Washington, US	N = 6	N = 0	N = 1	N = 2
3	Massachusetts Eye & Ear, Boston, Massachusetts, US	N = 2	N = 3	N = 3	N = 8
4	Nottingham University & Guys & St Thomas’ Hospital, London, UK	N = 0	N = 7	N = 0	N = 2
5	University Hospital Zürich, University of Zürich, Zürich, Switzerland	N = 0	N = 6	N = 0	N = 4
Total	n/a	N = 9	N = 20	N = 4	N = 20

* N = number of ears Electrode Contact Spacing
