# Supplementary material for: Assessing Array-Type Differences in Cochlear Implant Users Using the Panoramic ECAP Method
Source: Ear Hear. Author manuscript; Available in PMC 2025 Sep 1. (PMC7617747; doi:10.1097/AUD.0000000000001673)
Supplement: Supplementary file 1 [file EMS204296-supplement-Supplemental_Information.pdf]

Electrode Contact Spacing

Note: *All given dimensions are nominal*

Contour Advance (CI612), Electrode Spacing: 0.81 – 0.40mm

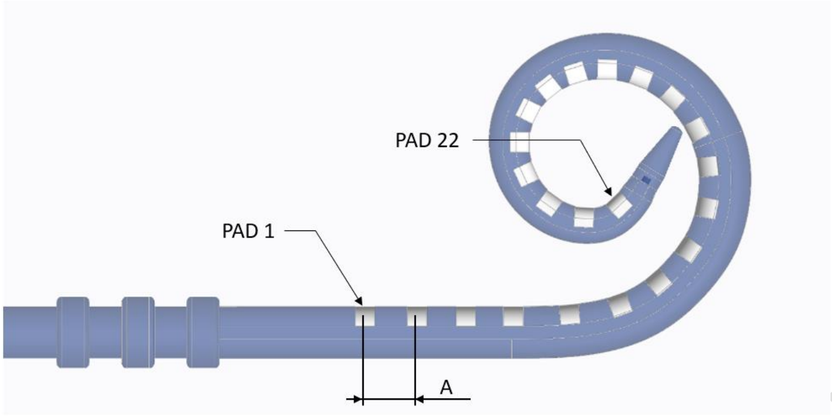

Slim Modiolar (CI632), Electrode Spacing: 0.60mm

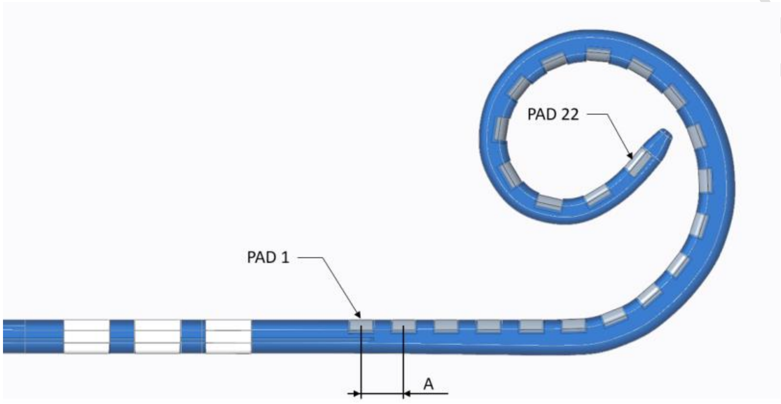

Slim Straight (CI622), Electrode Spacing: 0.85 – 0.95mm

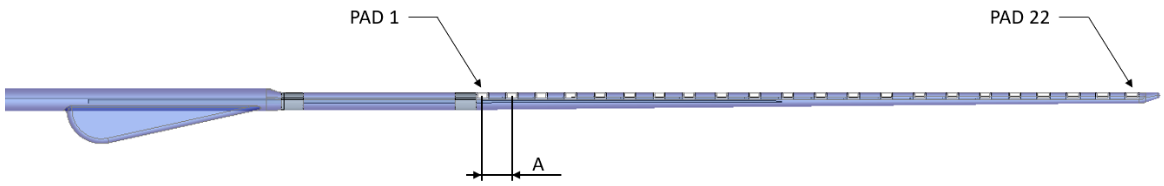

**Electrode Type**	**Electrode Contact Centre to Centre Distance (mm) (Dimension A)**
Contour Advance (CI612)	0.8 – 0.4
Slim Modiolar (CI632)	0.60
Slim Straight (CI622)	0.85 – 0.95

Information provided by Amanda Fullerton from Cochlear via email to Charlotte Garcia, 9 April 2024: "…there is slight variation in spacing, though it is not necessarily in a systematic basal-to-apical way. In addition, this spacing may change again once the electrode is inserted and curled inside the cochlea, depending on the degree of curvature."
